# Research on a Space–Time Continuous Sensing System for Overburden Deformation and Failure during Coal Mining

**DOI:** 10.3390/s23135947

**Published:** 2023-06-27

**Authors:** Gang Cheng, Zhenxue Wang, Bin Shi, Wu Zhu, Tianbin Li

**Affiliations:** 1 School of Computer Science, North China Institute of Science and Technology, Beijing 101601, China; chenggang@ncist.edu.cn (G.C.); wangzhenxue22@163.com (Z.W.); 2 School of Earth Sciences and Engineering, Nanjing University, Nanjing 210023, China; shibin@nju.edu.cn; 3 Key Laboratory of Ecological Geology and Disaster Prevention, Ministry of Natural Resources, Chang’an University, Xi’an 710054, China; 4 State Key Laboratory of Geohazard Prevention and Geoenvironment Protection, Chengdu University of Technology, Chengdu 610059, China; ltb@cdut.edu.cn; 5 Nanjing University High-Tech Institute at Suzhou, Suzhou 215123, China

**Keywords:** overburden deformation and failure, space–time continuous sensing, multi-algorithm processing, early warning

## Abstract

Underground coal mining can cause the deformation, failure, and collapse of the overlying rock mass of a coal seam. If the mining design, monitoring, early warning, or emergency disposal are improper, in that case, it can often lead to mining disasters such as roof falls, water inrush, surface collapse, and ground fissures, seriously threatening the safety of mine engineering and the geological environment protection in mining areas. To ensure the intrinsic security of the entire coal mining process, aspace–time continuous sensing system of overburden deformation and failure was developed, which breaks through the limitations of traditional monitoring methods that characterize the evolution process of overlying rock deformation and ground subsidence. This paper summarizes the classification of typical overburden deformation and failure modes. It researches the space–time continuous sensing of rock–soil mass above the coal seam based on Distributed Fiber Optic Sensing (DFOS). A multi-range strain optical fiber sensing neural series from micron to meter was developed to achieve synchronous sensing of overburden separation, internal micro–cracks, and large rock mass deformation. The sensing cable–rock mass coupling test verified the reliability of the optical fiber monitoring data. The sensing neural network of overburden deformation was constructed using integrated optical fiber layout technology on the ground and underground. Different sensing nerves’ performance and application effects in overburden deformation and failure monitoring were compared and analyzed with field monitoring examples. A physical model was used to carry out the experimental study on the overburden subsidence prediction during coal mining. The results showed that the optical fiber monitoring data were reliable and could be used to predict overburden subsidence. The reliability of the calculation model for overlying rock subsidence based on space–time continuous optical fiber sensing data was verified in the application of mining subsidence evaluation. A systematic review of the shortcomings of current overburden deformation observation technology during coal mining was conducted, and a space–time continuous sensing system for overburden deformation and failure was proposed. This system integrated sensing, transmission, processing, early warning, decision-making, and emergency response. Based on the fusion of multi-parameter sensing, multi-method transmission, multi-algorithm processing, and multi-threshold early warning, the system realized the real-time acquisition of space–time continuous information for the overburden above coal seams. This system utilizes long-term historical monitoring data from the research area for data mining and modeling, realizing the prediction and evaluation of the evolution process of overburden deformation as well as the potential for mining subsidence. This work provides a theoretical reference for the prevention and control of mining disasters and the environmental carrying capacity evaluation of coal development.

## 1. Introduction

**Figure 1:**
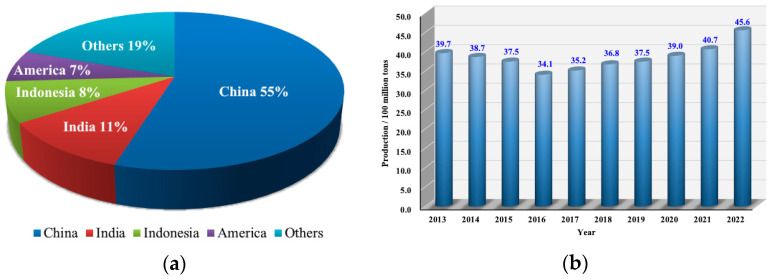
Distribution of coal production: (**a**) The proportion of global coal production in 2022; (**b**) China’s coal production from 2013 to 2022.

Coal resources are one type of energy mineral resource [1]. They are the most abundant and widespread conventional energy source in the world, accounting for 25% of global primary energy consumption. For a long time, China’s coal production has ranked first in global production, accounting for approximately half of the world’s coal production. The proportion of global coal production in 2022 and China’s coal production from 2013 to 2022 are shown in Figure 1.

**Figure 2:**
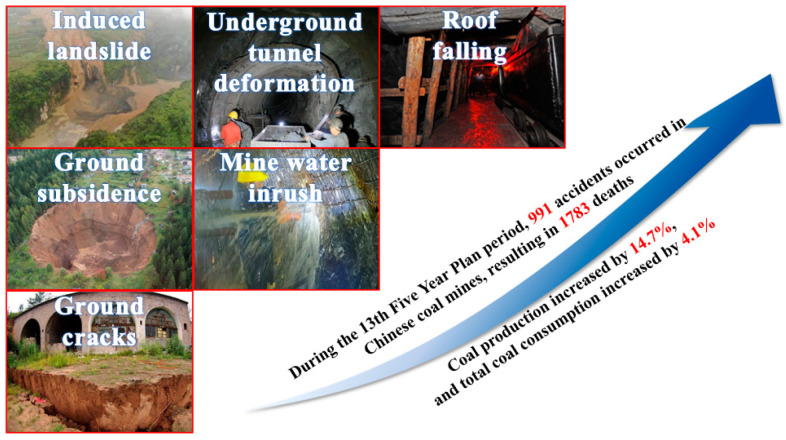
Mine disasters induced by coal mining.

In the past thirty years, the global economy’s rapid growth has resulted in an increase sharply in demand for energy consumption; among them, coal has become an important source of energy due to its rich reserves [2]. As shown in Figure 1a, China’s coal production ranked first in global coal production. With the depletion of shallow-buried coal resources, the mining depth increases continuously. According to relevant statistics recently, a large amount of coal production is achieved through underground mining in China, accounting for more than 90%. At present, India and Indonesia have a large proportion of open-pit mining, and the level of coal mine safety production is relatively high. With the increase in mining intensity, the coal seam is deepened, and the stripping ratio is increased, which leads to uneconomical mining. In the future, a part of coal resources will be converted into underground mining to improve coal production. Except for China, America has the most considerable underground mining output globally. It is one of the most advanced countries in underground mining technology. Underground mining significantly increases the difficulty of coal production. Improper mining technology and monitoring methods often cause mine disasters such as water inrush, underground tunnel deformation, roof falling, and ground cracks (Figure 2). For example, on 21 January 1960, a coal mine collapsed north of Coalbrook, South Africa, killing 435 miners and leaving no survivors. This was a relatively influential accident in the history of coal mining accidents. According to the Colombian National Mining Bureau, there were 1218 mining accidents in Colombia, resulting in 1306 miners being killed from 2011 to 2021. In China, on 14 August 2021, a major sand and mud inrush accident caused by roof caving occurred in the Qaidam Coal Mine of Qinghai Xihai Coal Development Co., Ltd., Haibei Tibetan Autonomous Prefecture, China, causing 20 deaths and direct economic losses of 53.9102 million CNY. On 25 February 2022, a severe roof collapse accident occurred in the Shunxun Coal Mine, Longchang Town, Zhenfeng County, Guizhou Province, China, resulting in 14 deaths and direct economic losses of 22.8847 million CNY. These disasters are often the results of overburden deformation, movement, and failure under mining, especially when closely related to the evolution characteristics of overlying strata [3,4,5,6], resulting in enormous casualties and economic losses, which seriously threaten mine safety production and ecological environmental protection. Therefore, preventing and controlling coal mining disasters and ensuring the safety of the whole process of mine engineering is a significant demand for global energy development. It can be seen that researching space–time continuous sensing of overlying rock deformation will provide data support for the accurate evaluation of coal seam safety mining and early warning prediction.

**Figure 3:**
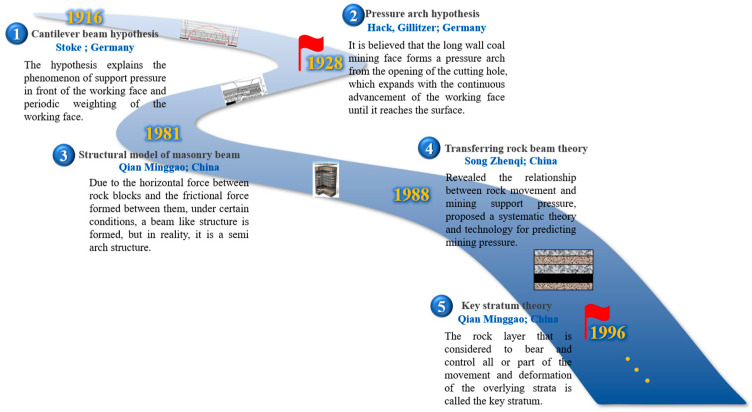
The development of the stope structure model.

Underground mining in coal mines often causes the deformation, separation, fracture, and collapse of overlying strata. Mine pressure is gradually created due to the movement of rock strata. In order to scientifically reveal the essence of mine pressure during mining, scholars at home and abroad have proposed a lot of hypotheses and theories of stope (Figure 3). In 1916, Stoke put forward the cantilever beam hypothesis, which explained the phenomenon of abutment pressure and periodic weighting of the working face. Hack and Gillitzer proposed a new pressure arch hypothesis in 1928 to explain the reason for surrounding rock unloading. After that, Kuznetsov proposed the hinged rock block hypothesis [7], which divided the failure of overlying strata in goaf into irregular caving zones, regular caving zones, and regular moving zones. In 1981, Chinese academician Qian Minggao proposed the theory of masonry beams [8,9] and established the overall mechanical model of stope [10,11]. The academician Liu Tianquan proposed the upper three zones theory of overburden failure [12]. In 1988, Academician Song Zhenqi proposed the transfer rock beam model [13], revealing the relationship between rock movement and mining abutment pressure. The same year, Chinese scholar Gao Yanfa put forward the four zones theory of rock movement [14]. In 1996, Qian put forward the key stratum theory [15], which realized the unity of research on mine pressure, strata movement, and water and gas migration. With the more profound study on the law of mine pressure, according to different geological conditions, scholars have proposed arch-like, arch–beam and beam [16], OX-, F-, T-type [17], and other overlying strata structure models, which provide an essential reference for improving the theory of overlying strata deformation.

However, due to the differences in the occurrence, development, and evolution of overlying rock deformation under different geological conditions, meanwhile, the deformation, movement, failure, and collapse of overlying strata is a continuous development process; the deformation both in vertical and horizontal of overlying strata and coal seams will change dynamically. Therefore, the deformation coordination and interaction between rock (soil) layers above the coal seam have a strong space–time effect. Most current studies have not considered this space–time continuous process, so they cannot fully reveal the core mechanism of overburden deformation during mining. Consequently, breaking through the technical bottleneck of coal mining monitoring is urgent. Thus, the space–time continuous cognition from overburden deformation and failure to surface-subsidence occurrence and development can be realized. The time-effect mechanism of overburden under coal mining was revealed. On this basis, a monitoring and guarantee system integrating sensing, transmission, processing, early warning, decision-making, and emergency was established based on an optical fiber neural sensing network, which is of great significance to the real-time intelligently sensing of overburden deformation, movement, failure, and collapse during coal mining.

## 2. Research on Space–Time Continuous Sensing of Overburden Deformation and Failure

### 2.1. Typical Modes of Overburden Deformation and Failure

**Figure 4:**
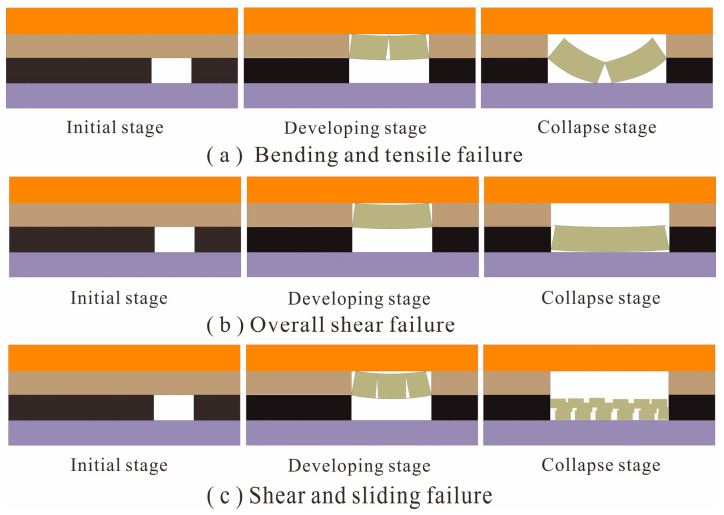
Typical types of overburden deformation and failure modes.

Global scholars have conducted many laboratory experiments and on-site tests about overburden deformation mechanism during coal mining. Systematic research has utilized similar material physical model tests, field in situ tests, numerical simulations, and theoretical calculations [18,19,20]. A more accurate understanding of the mode and evolution law of overburden deformation and failure was obtained. They are roughly divided into three categories: (a) bending and tensile failure; (b) overall shear failure; (c) shear and sliding failure [21]. For the fracture and separation evolution mode dominated by tension and bending (or compression), when the initial goaf is formed, the goaf upper strata bend due to the overburden pressure. With the goaf volume increasing, in the central section of the goaf, microcracks appear above the working face. The rock layer is fractured if the goaf reaches a certain length (Figure 4a). The conditions for tensile failure of roof rock layer *i* [22,23] are:

[σ_1_]_max_ > [σ], (1)

where [σ_1_]_max_ is the maximum principal stress of rock layer *i*, and [σ] is the tensile strength of rock layer *i*. Generally, the three-hinged arch structure is formed in the roof rock layer after failure. If the effective span *L* of the rock layer is greater than the limit span *L_i_* of the three-hinged arch structure, the three-hinged arch will collapse. Among them:

(2) $$L_{i}=\xi_{ic}\sqrt{0.04\frac{\sigma_{ic}a_{i}}{n\gamma \cos\beta }},$$

where $\sigma_{ic}$ represents the uniaxial compressive strength of rock layer *i*, $a_{i}$ is the fracture spacing of the weakest fracture group, $\xi_{ic}$ is the rock creep coefficient under compression, $\xi_{ic}$ = 0.5~0.7, n is the factor of safety, n = 4, and β is the weakest fracture inclination angle or dip angle of the rock layer.

Shear and slip failure often occurs near the excavation. It includes two types. One is fracture failure due to shearing action, which arises in the following situations: (a) the rock layer has high strength and stiffness; (b) the rock layer bears a tremendous shear force. Because the whole rock layer has high strength, cracks appear at both ends of the goaf. Under high shear stress, the rock layer will be cut off directly. At this moment, obvious cracks appear in the overlying rock. This is the leading reason resulting in mine disasters, which mainly occur in the brittle rock strata of the deep mining area (Figure 4b). The other is rock layer deformation under tension and bending. Overburden cracks are formed along both the horizontal and vertical directions, affected by the free-working face and the disturbance of the mining area. Then, cracks enlarge, close, and reappear again. Subsequently, due to compression and shearing, the rock layer undergoes segmented shearing and sliding, forming irregular caving gravel. The overlying strata usually experience slow and periodic damage at this stage (Figure 4c). According to the Mohr‒Coulomb criterion, the shear strength of rock layer *i* is:

[*τ*] = *C* + σ_α_tan*φ*, (3)

where [*τ*] is the shear strength of rock layer *i*, *C* is the cohesive of rock layer *i*, σ_α_ is the normal stress on sliding surface of rock layer *i*, and *φ* is the internal friction angle of rock layer *i*. Then, the condition of shear failure at point (x_0_, y_0_) is [24]:

*τ*_α_ ≥ [*τ*], (4)

where *τ*_α_ is the normal stress on the sliding surface of rock layer *i*.

Assuming Δ*τ* = *τ*_α_ − [*τ*], if Δ*τ* takes the maximum value [Δ*τ*]_max_ at point (x_0_, y_0_), then point (x_0_, y_0_) is the most susceptible to shear failure in the rock layers.

### 2.2. Space–Time Continuous Sensing Based on DFOS Technology

#### 2.2.1. The Principle of DFOS Technology

**Figure 5:**
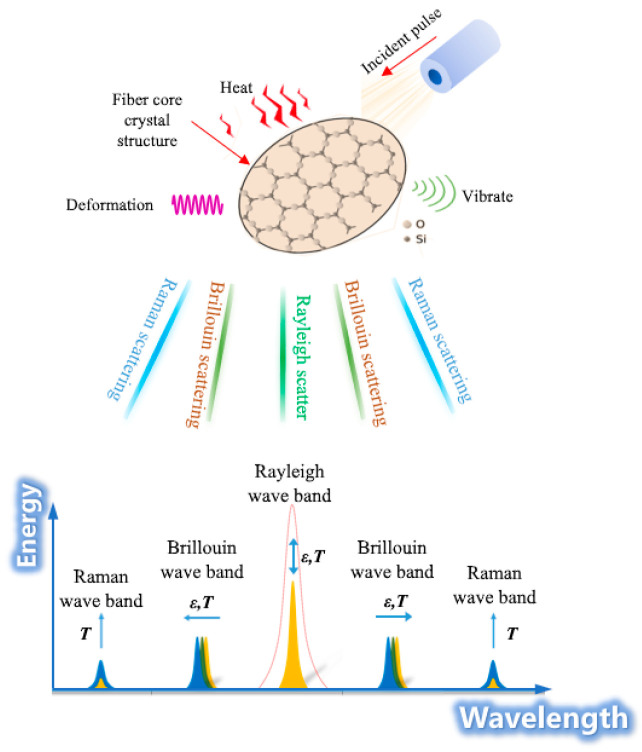
Principle of fiber optic sensing technology [30].

**Figure 6:**
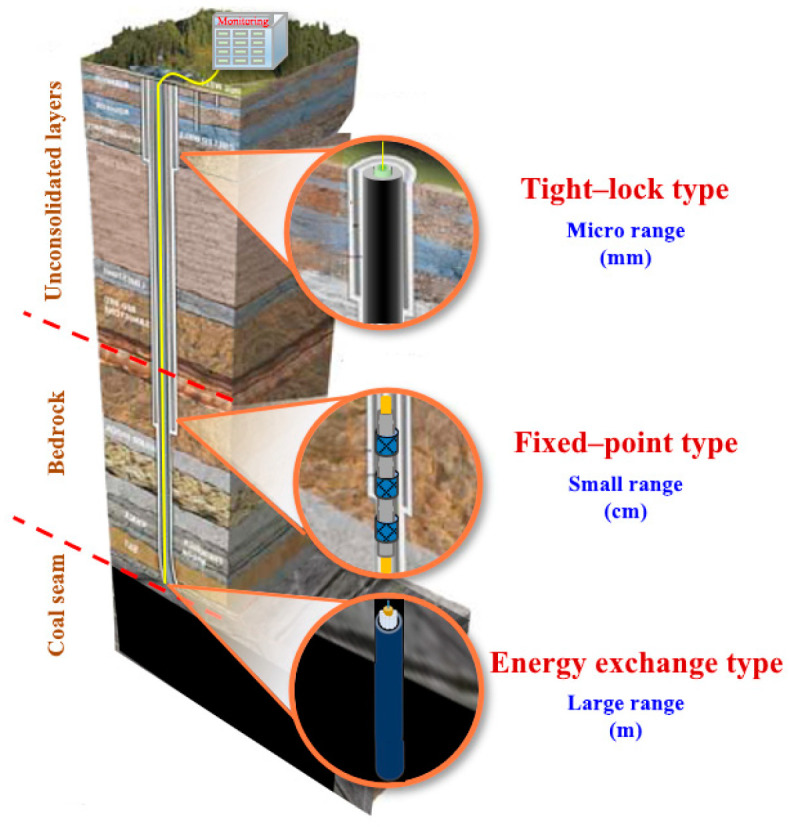
A series of multi-range strain sensing neural cables.

Fiber optic sensing technology integrates the advantages of both optical sensing and data transmission. By using the change in the scattered light characteristic quantity in the optical fiber caused by external factors, the related physical amount of the measured object along the optical fiber can be continuously monitored for a long distance [25] (Figure 5). In the latest twenty years, the application field of fiber optic sensing technology has constantly expanded, and the types of sensing cables are also increasing [26,27,28]. However, rock–soil mass will undergo multi-scale deformation in applications. It is necessary to fully consider the conditions of the monitoring object and select the sensing cable according to its characteristics [29]. Fixed-point homogenization and equivalent energy conversion have been invented, forming a series of multi-range strain sensing neural cables from the micron to the meter (Figure 6).

#### 2.2.2. Sensing Cables and Sensors in Mine Engineering Monitoring

**Figure 7:**
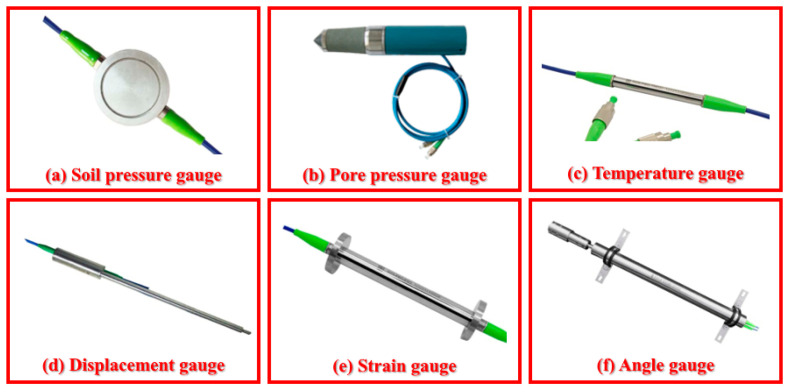
Fiber optic sensors for mine engineering monitoring.

According to the characteristics of mine engineering monitoring, glass fiber-reinforced plastic (GFRP), metal-based, and fixed-point cables are commonly used. The GFRP cable adopts high-strength polyurethane as the reinforcement, which not only ensures a high strain transfer performance, but also makes it resistant to tension, compression, and impact during the test. Therefore, it is widely used in deformation, internal force, and damage monitoring in mine engineering. The metal-based cable adopts a particular technique of steel wire twisting and packaging, which has strong wear resistance and ensures its survival rate under high-pressure grouting and pouring backfilling. It is suitable for the deformation monitoring of materials with a high elastic modulus (bedrock, concrete, etc.). The fixed-point cable calculates the deformation of the measured target by using the strain value between its two fixed points, which is suitable for discontinuous and large deformation monitoring of rock–soil mass. The overburden separation and internal microcracks can be accurately identified based on the high-sensitivity cable between two contiguous fixed points. Different cables’ parameters are shown in Table 1. The author’s research team and the R&D institution developed a series of fiber optic sensors using fiber Bragg grating (FBG) technology (Figure 7), suitable for geological and geotechnical engineering monitoring. They can meet the dense monitoring of key positions in overburden rock and soil, as well as achieve complementary verification with DFOS technology.

#### 2.2.3. The Cable–Rock Mass Coupling Test

**Figure 8:**
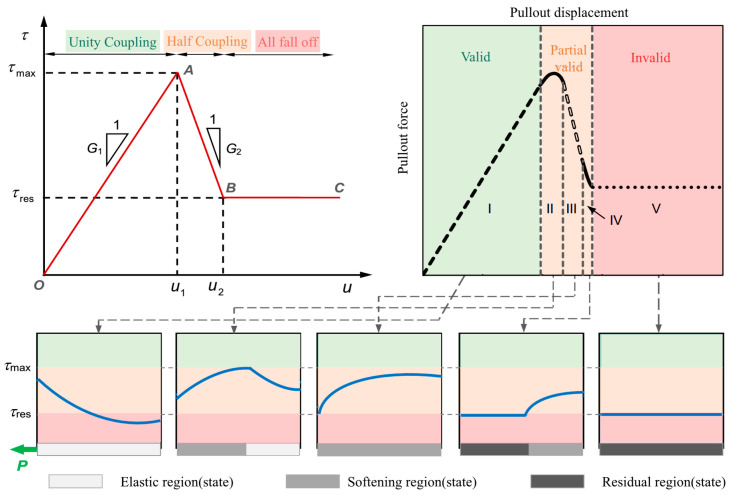
Simplified criterion of sensing cable monitoring accuracy.

**Figure 9:**
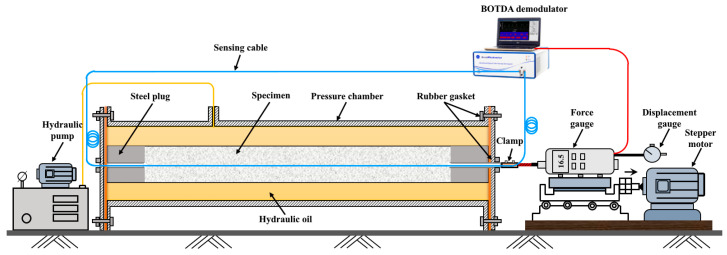
Coupling test device for cable–rock mass under controllable confining pressure.

The coupling performance between the sensing cable and the rock–soil mass affects the quality of measured data, significantly impacting the reliability of the monitoring results [31]. Therefore, the cable–rock mass coupling problem is a critical problem that must be solved in the distributed monitoring of rock–soil mass deformation in mine engineering. In the past decade, global scholars have researched the coupling performance of cable–rock mass through theoretical calculations, pull-out tests, and field measurements. Zhu Honghu and Cheng Gang investigated fiber-sand coupling through the pull-out test based on Brillouin optical time-domain analysis (BOTDA) technology. They studied the coupling process and analyzed the distribution law of strain along the optical fiber. Then, the interaction between the optical fiber and rock was divided into three stages: (a) unity coupling; (b) half coupling; (c) all fall off. The established relationship model for displacement is shown in Figure 8 [32]. Professor Shi Bin’s research group at Nanjing University systematically researched the coupling between the sensing cable and the loose sand, compacted sand, and clay–mixed soil under different confining pressures by developing a controllable confining pressure cable–rock mass interaction pull-out test device (Figure 9) [33,34]. It was concluded that the interface between cable and rock presents progressive failure characteristics under the pull-out state and is closely related to the confining pressure level.

**Figure 10:**
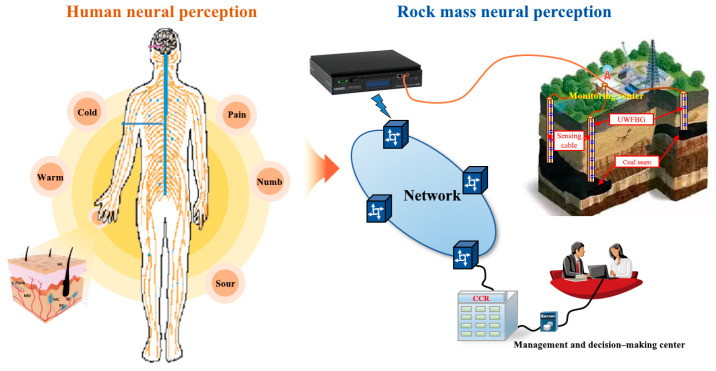
Distributed neural sensing system for rock–soil mass.

In recent years, more and more monitoring methods have been used to investigate the failure law of overlying rock and the evolution process of mining subsidence. Traditional sensing elements such as resistance strain gauges, displacement gauges, osmometers, temperature gauges, and electrodes are primarily used in the laboratory and on-site tests. Although these monitoring technologies can obtain the strain, displacement, seepage pressure, temperature, resistivity, and other physical parameters of the local position of the overlying rock–soil mass on the coal seam, it is generally found that the measurement accuracy of the sensing element is low, the layout is cumbersome, the durability is poor, and the data are discrete. It is impossible to comprehensively track the cumulative deformation of the overlying strata induced by continuous mining [35]. Obtaining multi-source and multi-field information in the underground rock–soil mass is difficult. Determining the cumulative mining subsidence of overlying strata has become an additional problem. With the continuous development of distributed fiber optic monitoring theory, technology, and application, a revolutionary breakthrough has been made in the analysis theory and test method of overburden deformation during mining. Its unique advantages, such as distributed, high-precision, long-distance, fully real-time, anti-interference, and small in size, enable real-time acquisition of physical information (strain, temperature, vibration, et al.) at all locations in the length direction of an optical fiber (cable) [36]. On this basis, Professor Shi Bin of Nanjing University proposed an earth-sensing theory (Figure 10) [37]. The sensing cable is arranged in a network according to the demand, which is similar to implanting a sensing nerve into the overlying strata and perceiving every action of the overlying strata at all times.

#### 2.2.4. The Cable Layout for Ground and Underground Monitoring

**Figure 11:**
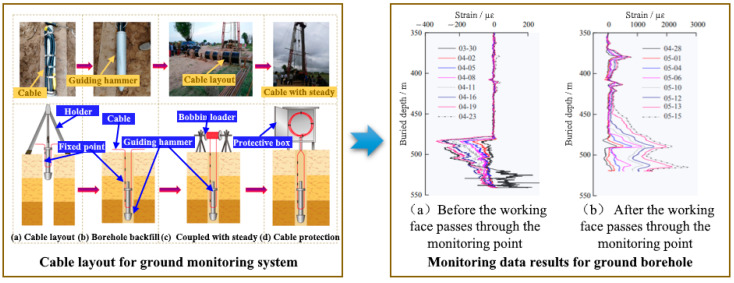
Cable layout and data results for ground monitoring system [38].

Many research results show that the layout process and quality of the monitoring system directly influence the monitoring results. Therefore, it is essential to select the corresponding layout technique according to different test environments and objects in the safety mining monitoring of mine engineering. Currently, most test systems combine ground with underground techniques in the practical application of mine engineering. The surface layout method is mainly the ground monitoring system layout. Firstly, implement a monitoring borehole on the ground. Then, according to the different monitoring conditions and geological environment, it is essential to select the appropriate sensing cable and device for the borehole layout. There are four stages: (a) making a counterweight guiding hammer; (b) implanting a sensing cable; (c) borehole backfilling coupled with steady; (d) sensing cable protection (Figure 11).

**Figure 12:**
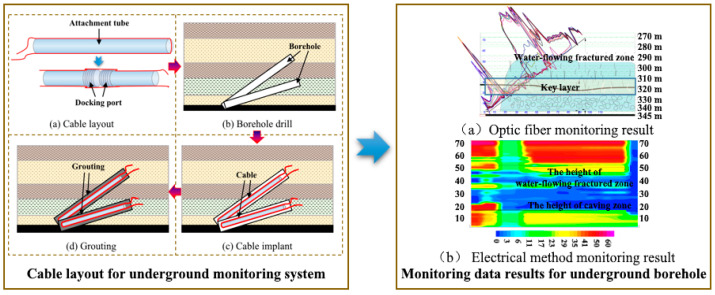
Cable layout and data results for underground monitoring systems [39].

The underground layout method mainly uses high-strength lightweight pipe fittings as the sensing cable (sensor) attachment carrier, which is buried in the rock mass above the coal seam. Firstly, the underground upwards borehole is designed according to the stope conditions. Secondly, Implement a monitoring borehole above the working face with a certain angle. Meanwhile, cable layout and grouting operations are carried out. Finally, the initial value is collected after the borehole reaches the coupling strength. Regularly collect monitoring data based on the actual mining progress of the working face. The response characteristics of overburden deformation and failure in the borehole control height under mining action are analyzed based on the initial value of the sensing cable and the regular data. Zhang Pingsong et al. carried out a dynamic monitoring test for roof overburden combining optical and electrical technology, designed a set of monitoring sections, implemented two roof boreholes at depths of 150 m and 135 m, controlled the advance distance of 96 m, controlled the vertical height of 70 m, and obtained a caving zone height of 18.5 m and water–flowing fractured zone height of 54.5 m (Figure 12). The results show that the resolution and recognition accuracy can be improved based on the optical and electrical parameters.

Based on the mode distribution characteristics of overlying rock deformation and failure, the space–time continuous strain data of overlying rock can be obtained using fiber optic sensing technology, which can accurately grasp the mining progress and conditions. The stress concentration and electric field (resistivity) mutation areas are captured, combined with the variation law of the geothermal gradient, pore water pressure, and overburden resistivity, which can infer the evolution stage and potential failure mode of overburden stability. At the same time, the early warning analysis of periodic anomalies, multi-parameter common anomalies, and the statistical analysis of historically accumulated data should be strengthened. The early warning model based on critical indicators such as deformation, temperature, seepage pressure, and resistivity should be researched to improve the early warning accuracy of overlying rock deformation during coal mining.

### 2.3. Applied Case Analysis

To study the evolution mechanism of deformation and failure for overburden, providing a theoretical basis for disaster prevention and control of mining subsidence, Liu Yu [40] et al. researched the deformation characteristics of overlying rock under mining utilizing Brillouin optical time-domain reflectometer (BOTDR) technology. Different sensing cables can reflect different sensing parameters and properties. Considering the optical parameters, tensile strength, and transmission coefficient of the cables, selected three kinds of cables: the metal kieso sensing (MKS) cable, glass fiber-reinforced sensing (GFRS) cable, and 10 m interval fixed-point sensing (10 m IFS) cable for on-site test. The detailed layout procedures for on-site test mainly includes: (a) Fix the cables on the guiding cone with adhesive; (b) Clean the borehole before burying; (c) Connect the guiding cone with the drill pipe by a thread device; (d) Implant the cables into the borehole at a steady lowering speed; (e) Once the guiding cone gets to the hole’s bottom, use the spiral action of the drilling machine to separate the guide cone from the drill pipe; (f) Grout in the rock formation with a special mixture and backfill with sand and soil with similar properties in unconsolidated layers; (g) Seal the borehole, and carefully protect the interface of the cables. The on-site test results indicated that the sensing performance of the MKS cable was the best. During coal mining monitoring, the strain on the upper part of the cable was from compression to tension. In contrast, on the lower part of the cable was a process of compression to tension and then to compression. This may impact the vertical deformation features of the overlying rock. Meanwhile, the deformation consistency of the sensing cable and backfill material, backfill material and surrounding rock were studied respectively. As a result, the surrounding rock and sensing cable’s consistency of deformation were assessed. From the overall trend of test results, the monitored deformation was smaller than the actual deformation of the backfill material, and as the displacement increased, the gap typically enlarged. Overall, the cable’s strain had a good correspondence with the overburden deformation. Through the above test analysis, compared to conventional monitoring techniques, BOTDR technology can more accurately measure the dynamic height of the fractured zone. This has major implications for the monitoring of overburden deformation and failure during coal mining, preventing water inrush, and ground ecological geological disasters.

**Figure 13:**
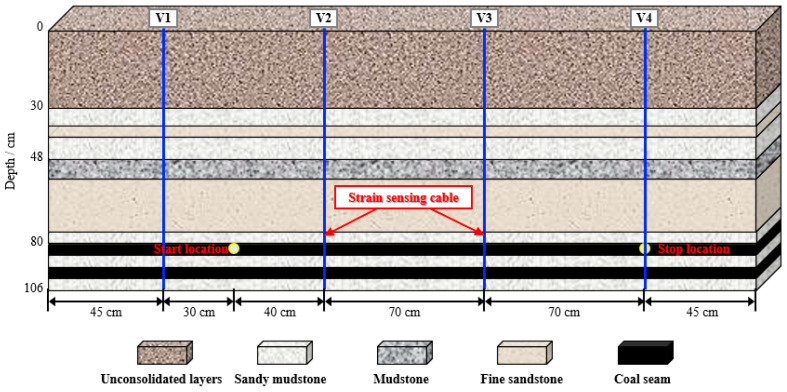
Optical fiber layout in the model test of predicting the subsidence.

Piao Chunde [41] predicted the overburden subsidence under coal mining based on DFOS technology, then evaluated the surface stability according to the monitoring data. Figure 13 depicts the model test’s optical fiber arrangement. The coal seam’s mining thickness was 42 cm, and each area’s mining width was 5 cm. Four sensing cables (V1, V2, V3, V4) were laid along the vertical direction of the model to grasp the failure characteristics of the overlying rock in the coal mining process, which were 45 cm, 115 cm, 185 cm, and 255 cm from the left side of the model, respectively. Depending on the gray theory and Knothe time function, a subsidence prediction model of mining overburden based on measured strain was established, which can accurately reflect the development characteristics of the caving zone and fractured zone. It was suitable for the early stage and later stages of overburden subsidence. He studied the state of the overlying strata above the goaf to establish a subsidence calculation model for overburden, which was based on rock mechanics parameter theory and BOTDR technology. On this basis, considering the effects of contact parameters and rock damage distribution, it was obtained about the space–time distribution law of overburden subsidence. Taking the Zhanghuaizhu working face of the Zhangzhuang Coal Mine as the investigated target, there was a negative exponential relationship between the subsidence in the goaf with time. The trend of surface subsidence was the same. The calculation model’s relative error was lower than the regional monitoring value’s error, which was less than 8%. Therefore, it is reliable to calculate the subsidence value of the overburden in the goaf with the subsidence calculation model.

## 3. The Development Trend

**Figure 14:**
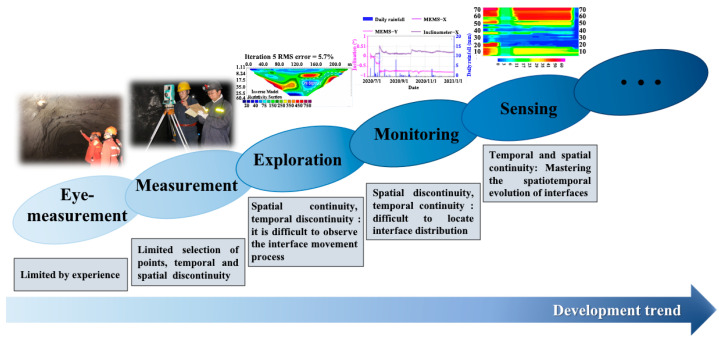
Development trend of overburden deformation and movement observation technology.

For a long time, global scholars have researched the occurrence, development, and failure characteristics of overburden deformation during mining through empirical formulas, mechanical analysis, numerical calculation, model tests, and field detection. Karmis M (1983) and Palchik (2002) found that there were three different moving zones (caving zone, fractured zone, continuous deformation zone) above the coal seam under the condition of longwall mining through field measurement [42,43]. Based on previous research experiences and results, Chinese scholars have analyzed and summarized the evolution laws of water–flowing fractured zone under conventional mining conditions, putting forward a calculation formula to calculate the development height of the water–flowing fractured zone in three kinds of underground mining (under buildings, railways, and water bodies) [44]. Li Quansheng et al. (2022) studied the fracture evolution law from the formation process, research methods, and monitoring means of mining fractures [45]. Chai Jing et al. (2012) characterized rock–soil mass deformation by implanting FBG into boreholes [46]. Wang Huiqiang, Zhu Jianjun et al. (2019) proposed the parametric modeling method of motion errors to improve InSAR measurement accuracy [47]. Xie Heping (1999) and Xu Yanchun (2002) proposed the separation prediction method according to the structure types of overlying strata [48,49]. Zhang Pingsong et al. (2011, 2019) monitored overburden deformation through the borehole resistivity method [50,51]. The above methods are essential for studying the deformation mechanism of overlying strata. However, the observation techniques of mining-induced overlying strata deformation and movement are divided into five categories: eye–measurement, measurement, exploration, monitoring, and sensing (Figure 14), from the space–time continuous deformation information and the observation results. There are the following shortcomings in the study of overburden deformation and failure mechanisms: (a) Eye–measurement technology is space–time discontinuous and depends on surveyors’ experience; (b) Measurement and exploration technology are discontinuous, and obtaining real-time state information of overlying strata in coal seams is difficult; (c) Monitoring technology is mostly point type, with discrete and discontinuous data in space. It is impossible to fully track the cumulative deformation of overburden under continuous mining. At the same time, the geological conditions of each mining area are quite different, and the empirical formula is one-sided. The mechanical analysis must be compared and corrected with reliable long-term observation data. The randomness of parameter selection criteria in the numerical calculation is significant, and there are some differences between different software results. The model test is challenging to simulate coal mining with complex geological structures effectively.

**Figure 15:**
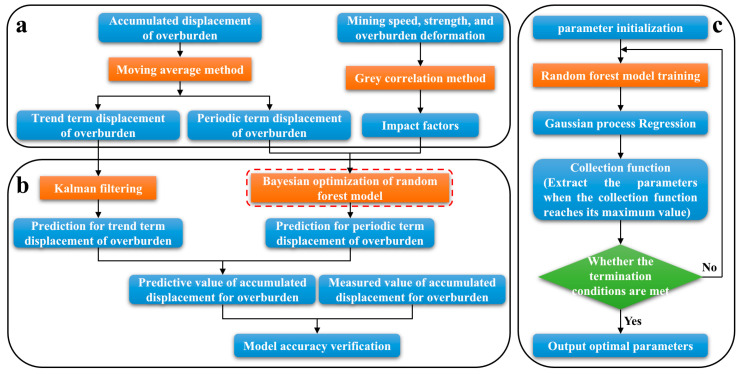
Intelligent processing and algorithm optimization of massive data [52]. (**a**) Process for determining overburden displacement and its influencing factors; (**b**) Model prediction accuracy verification; (**c**) Process for random forest model training.

In particular, clustering, regression analysis, Bayesian inference, genetic algorithms, and gray theory can be introduced into the integrated overburden deformation sensing system for data mining and modeling with the rapid development and broad application of machine learning, chatGPT, and other technologies. Mining out the disaster characteristic information can reflect the overlying rock deformation and instability during coal mining. The temporal information, spatial information, multi-modulus, and other attributes of monitoring data were used to construct the hypergraph structure of big data for overburden deformation. Hypergraph clustering and segmentation technology were used to complete big data mining and prediction of overburden deformation. Bayesian rules were used to optimize feature information mining and prediction results. According to the strain evolution characteristics of overburden, combined with the variation laws of geothermal gradient, pore water pressure and overburden resistivity, the stress concentration area and electric field (resistivity) mutation area were captured, and the evolution stage and potential failure mode of overburden stability were inferred accordingly. The primary processes of this method are as follows: Through a data cleaning method based on the topic model and integrating the effectiveness analysis mechanism of the IoT sensing data source, intelligent processing of massive data in the whole process of overburden deformation and failure sensing is carried out. These strengthened the machine learning research of key layer data missing from error correction, association analysis, and data mining. The hyperparameters were optimized by combining optimization algorithms such as moving average, random forest, Kalman filter, and multi-variate Bayesian. The trend term displacement of overburden was predicted based on the Kalman filter. The Bayesian optimized random forest model was used to predict the periodic term displacement of overburden, and the predictive value of accumulated displacement of overburden was formed. The model’s accuracy was verified and combined with the measured data of displacement to improve the prediction accuracy of overburden deformation (Figure 15).

**Figure 16:**
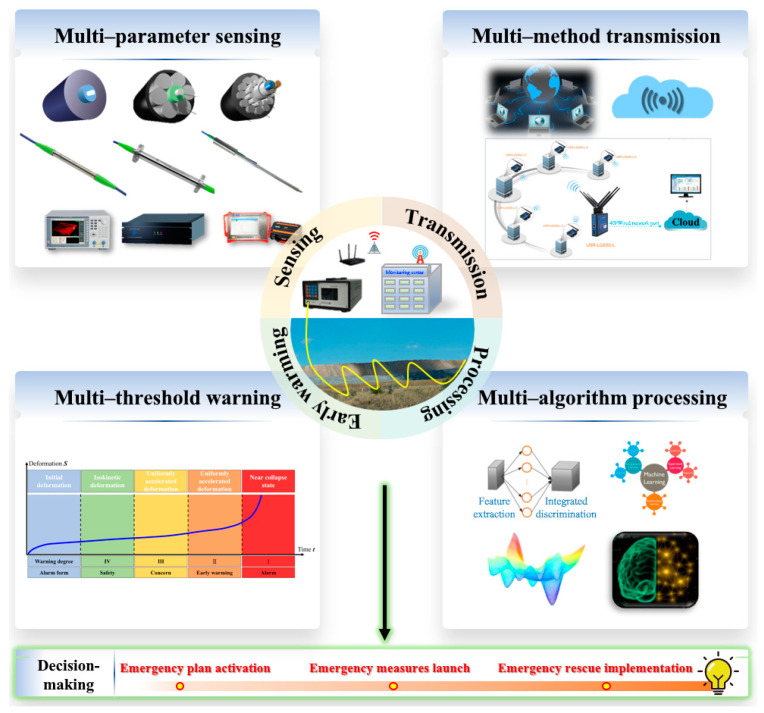
Integrated system for overburden deformation sensing.

The current methods are difficult to continuously obtain the impact of overlying rock–soil mass deformation during coal mining on local deformation of underground tunnels and ground subsidence. Therefore, it is vital to overcome the technical barriers of sensing massive space–time continuous information in overburden during coal mining and carry out space–time continuous sensing research on the evolution from overlying rock deformation and failure to surface subsidence. Based on this, an integrated safety guarantee system for coal mining that covers sensing–transmission–processing–early warning–decision is established (Figure 16): (a) A multi-parameter sensing device converted the physical field state information of the rock–soil mass into the sensor photoelectric signal, which was sent to the demodulation device for a series of signal processing and calibration steps. R&D of multi-source and multi-parameter sensing cables are needed to realize the distributed, continuous, and large-scale real-time acquisition of multi-field data of rock–soil mass, promoting overburden deformation monitoring technology to innovate in the direction of a wider detection range, deeper depth, and higher data transmission efficiency. The monitoring content is developing towards diversification, precision, and three-dimensionality; (b) The critical technologies of the transport layer include wired transmission and short-range wireless communication technology. Because most mining engineering has a wide range, strong concealment, poor monitoring environment, and high dynamic response requirements, it is often in the area without a communication network signal in the actual monitoring process. Therefore, the corresponding transmission technology should be selected according to the monitoring target and environment. A wireless sensor network (WSN) can be used to improve the monitoring accuracy of overburden deformation and mining subsidence; (c) During coal mining, abnormal mutation and disorder of optical fiber monitoring data often occur owing to the fracture and collapse of overlying strata, especially sudden overall collapse. Therefore, it is essential to denoise and filter the optical fiber data to restore the overburden deformation and failure process. On this basis, mathematical statistics, artificial intelligence theory, and systematic methods were introduced to optimize the preprocessing algorithm for classifying and extracting massive sensing data to improve data reliability, self-diagnosis, and intelligent analysis accuracy; (d) Monitoring is only a means, and early warning is the purpose. In the deformation monitoring of mining overburden, the corresponding monitoring content, specific evaluation index, and scientific early warning criterion should be designed according to factors such as strata lithology, mining height and rate. Meanwhile, it is essential to strengthen the differential research on warning thresholds, conducting differential warning thresholds and levels investigations based on different factors such as the material composition, key stratum thickness, and mining rate. It can improve the matching degree between the threshold and the evolution process of strata movement and minimize the missed and false alarm rates. Based on the analysis and processing results of the sensing–transmission–processing–warning layers, emergency decision-making is made to achieve an accurate assessment of coal mining disaster risk as well as prediction and evaluation of ground subsidence potential.

## 4. Conclusions

This paper summarized the typical types of overburden deformation and failure modes. It compared and analyzed the parameter performance and applicable conditions of sensing cables (sensors) suitable for overburden deformation monitoring in mine engineering. Based on the achievements of the author’s research team in the coupling test for sensing cable–rock mass, a high-precision, large-capacity, and full-coverage distributed sensing network for mining was constructed, combined with the ground–underground integrated cable layout techniques. A space–time continuous sensing system for the overburden deformation and failure integrating sensing–transmission–processing–warning–decision–emergency was proposed. The main conclusions are as follows:

(1) There are three typical modes of tensile failure, overall shear, and shear slip in the overburden deformation and failure induced by mining. Their occurrence, development, and evolution have a cumulative space–time effect. To clarify the evolution process and disaster mechanism of overburden deformation and failure, research on the space–time continuous state sensing for overlying strata should be strengthened, the space–time continuous sensing data of overburden deformation and failure should be integrated, and the accurate characterization of overburden deformation and failure should be realized. It is necessary to focus on the abrupt abnormal data, consider the gradual change data of overburden deformation regularity simultaneously, and realize the scientific evaluation for the stability of overlying strata during mining by combining the distribution of stratum profile and the field measured data.

(2) According to the characteristics of mine engineering monitoring, combined with the performance parameters and application conditions of the sensing cables (sensors) used for overburden deformation monitoring, a multi-range strain optical fiber sensing nerve series from micrometer to meter is formed to realize the simultaneous sensing of overburden separation layer and its internal microcracks as well as large deformation of the overlying rock. The testing method, process, and criteria for the cable–rock coupling test to verify the reliability of optical fiber monitoring data were introduced. By utilizing the integrated method of laying cables on the ground and underground, the installation process of cables has been improved, and a distributed fiber optic neural sensing network has been set up to achieve space–time continuous and precise sensing for the overburden deformation during the whole cycle of coal production. In particular, for the large deformation monitoring of mines, it was proposed to reduce the diameter of the fixed-point cable’s outer sheath, and adapt the shape of the fixed point to be an olive shape, which can not only avoid scratching the sensing cable during cable layout but can also improve the coupling performance between the cable and the rock–soil mass.

(3) Based on DFOS technology, a space–time continuous sensing system for the deformation of rock–soil mass above the coal seam during mining was constructed, integrating multi-parameter sensing, multi-method transmission, multi-algorithm processing, and multi-threshold warning. The real-time sensing of space–time continuous information under mining was realized. The disaster risk of coal mining and the potential of ground subsidence was accurately predicted by combining computer intelligence analysis algorithms such as clustering, regression analysis, Bayesian inference, genetic algorithm, and gray theory to realize the whole process cognition from the deformation, failure, and collapse of overburden to the occurrence, development, and evolution of ground subsidence.

## Figures and Tables

**Table 1:** The parameter summary for different types of sensing cables.

Cable Type	Diameter (mm)	Tensile Strength (N)	Range	Unit Weight (kg/km)	Characteristics
GFRP Cable 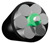	5.8	3000	−1~1%	28	Fine measurement, low shear strength and wear resistance, high requirements for layout environment.
Metal-based cable 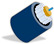	5.0	3500	−1~1%	38	High shear strength, wear resistance, good environmental adaptability, and general measurement accuracy.
Fixed-point cable 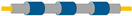	5.0	1000	0~3%	36	The measurement range is extensive and can reach the meter level, suitable for segmented fine monitoring of large deformation.
Steel wire armored temperature cable 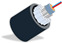	8.0	1800	−40~85 °C	45	Good environmental adaptability, strong anti-damage ability, and general temperature sensitivity.
Plastic armored temperature cable 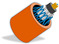	3.0	400	−20~85 °C	27	It has good coupling with the rock and soil, high sensitivity for temperature, general tensile capacity, and a narrow temperature tolerance range.

## Data Availability

Not applicable.

## References

[1] Peng S.P. (2009). China coal resource exploitation and environmental protection. Sci. Technol. Rev..

[2] Huang Q.X. (2008). Green mining of coal resources. Shaanxi Coal.

[3] Zhang T., Gan Q., Zhao Y.X., Zhu G.P., Nie X.D., Yang K., Li J.Z. (2019). Investigations into mining-induced stress–fracture–seepage field coupling effect considering the response of key stratum and composite aquifer. Rock Mech. Rock Eng..

[4] Liu H.H., Zheng L.G., Zheng J.T. (2015). Relationships between permeability, porosity and effective stress for low-permeability sedimentary rock. Int. J. Rock Mech. Min. Sci..

[5] Cheng G., Shi B., Zhu H.H., Zhang C.C., Wu J.H. (2015). A field study on distributed fiber optic deformation monitoring of overlying strata during coal mining. J. Civ. Struct. Health Monit..

[6] Shi B. (2013). On field and their coupling in engineering geology. J. Eng. Geol..

[7] Peng W.Q. (2006). Study on the Failure of Overlying Rock in Mining Shallow Thick Coal Seam by Slicing Method. Master’s Thesis.

[8] Qian M.G. (1981). Conditions required for equilibrium of overlying strata at working areas. J. China Inst. Min. Technol..

[9] Qian M.G. (1981). A study of the behavior of overlying strata in longwall mining and its application to strata control. Dev. Geotech. Eng..

[10] Qian M.G., Zhang D.L., Li L.J., Kang L.X., Xu J.L. (1994). “S–R” stability for the voussoir beam and its application. Ground Press. Strat. Control.

[11] Qian M.G., Zhu D.R., Wang Z.T. (1986). The fracture type of main roof and their effects on roof pressure in coal face. J. China Inst. Min. Technol..

[12] Beijing Mining Research Institute of Chinese Coal Science Research Institute (1981). The Laws of Ground Subsidence Andoverburden Failure and It’s Applications on Coal Mines.

[13] Song Z.Q. (1988). Practical Mine Pressure Control.

[14] Gao Y.F. (1996). “Four-zone” model of rockmass movement and back analysis of dynamic displacement. J. China Coal Soc..

[15] Qian M.G., Miao X.X., Xu J.L. (1996). Theoretical study of key stratum in ground control. J. China Coal Soc..

[16] Jiang F.X., Song Z.Q., Song Y. (1993). Basic structure forms of main roof. Chin. J. Rock Mech. Eng..

[17] Dou L.M., He H. (2012). Study of OX–F–T spatial structure evolution of overlying strata coal mines. Chin. J. Rock Mech. Eng..

[18] Chai J., Ouyang Y.B., Liu J.X., Zhang D.D., Du W.G., Lei W.L. (2021). Experimental study on a new method to forecasting goaf pressure based key strata deformation detected using optic fiber sensors. Opt. Fiber Technol..

[19] Chai J., Du W.G., Yuan Q., Zhang D. (2019). Analysis of test method for physical model test of mining based on optical fiber sensing technology detection. Opt. Fiber Technol..

[20] Zhu L., Gu W.Z., Pan H., Liu Z.C., Chai J., Ouyang Y.B. (2021). Calculation model of overburden rock failure volume in mined-out area based on Brillouin optical time-domain analysis technology. Opt. Fiber Technol..

[21] Cheng G., Xu W.T., Shi B., Wu J.H., Sun B.Y., Zhu H.H. (2022). Experimental study on the deformation and failure mechanism of overburden rock during coal mining using a comprehensive intelligent sensing method. J. Rock Mech. Geotech. Eng..

[22] Xu J.L., Qian M.G. (2000). Method to distinguish key strata in overburden. J. China Univ. Min. Technol..

[23] Shi H., Jiang F.X. (2004). Mechanical analysis of rupture regularity of hard and massive overlying strata of longwall face. Chin. J. Rock Mech. Eng..

[24] Jiang Y.D., Yang Y.M., Ma Z.Q., Li Y.W. (2016). Breakage mechanism of roof strata above widespread mined-out area with roadway mining method and feasibility analysis of upward mining. J. China Coal Soc..

[25] Shi B., Zhang D., Zhu H.H., Zhang C.C., Gu K., Sang H.W., Han H.M., Sun M.Y., Liu Y. (2021). DFOS applications to geo-engineering monitoring. Photonic Sens..

[26] Zhu H.H., Liu W., Wang T., Su J.W., Shi B. (2022). Distributed acoustic sensing for monitoring linear infrastructures: Current status and trends. Sensors.

[27] Zhang L., Shi B., Zeni L., Minardo A., Zhu H.H., Jia L.X. (2019). An Fiber Bragg Grating-based monitoring system for slope deformation study in geotechnical centrifuge. Sensors.

[28] Zhang L., Cui Y.F., Zhu H.H., Wu H., Han H.M., Yan Y., Shi B. (2023). Shear deformation calculation of landslide using distributed strain sensing technology considering the coupling effect. Landslides.

[29] Zhang L., Zhu H.H., Han H.M., Shi B. (2023). Fiber optic monitoring of an anti-slide pile in a retrogressive landslide. J. Rock Mech. Geotech. Eng..

[30] Shi B., Zhu H.H., Zhang C.C., Sun M.Y., Zhang W., Zhang T.Y. (2023). Rock and soil disaster sensing and application. Sci. Sin. Technol..

[31] Zhang C.C., Shi B., Zhang S., Gu K., Wei G.Q. (2021). Microanchored borehole fiber optics allows strain profiling of the shallow subsurface. Sci. Rep..

[32] Cheng G., Wang Z.X., Zhu H.H., Li D.Y., Xu W.T., Zhang L. (2022). Experimental study on overlying strata deformation and failure using distributed intelligent sensing. advances in geoengineering along the belt and road. BRWSG 2021. Lect. Notes Civ. Eng..

[33] Zhang C.C., Shi B., Gu K., Liu S.P., Wu J.H., Zhang S., Zhang L., Jiang H.T., Wei G.Q. (2018). Vertically distributed sensing of deformation using fiber optic sensing. Geophys. Res. Lett..

[34] Zhang L., Cheng G., Wu J.H., Minardo A.D., Song Z.P. (2021). Study on slope failure evolution under surcharge loading and toe cutting with BOTDA technology. Opt. Fiber Technol..

[35] Horiguchi T., Shimizu K., Kurashima T., Tateda M., Koyamada Y. (1995). Development of a distributed sensing technique using Brillouin scattering. J. Lightwave Technol..

[36] Zhu H.H., Shi B., Zhang J., Yan J.F., Zhang C.C. (2014). Distributed fiber optic monitoring and stability analysis of a model slope under surcharge loading. J. Mt. Sci..

[37] Shi B. (2017). On the ground sensing system and ground sensing engineering. J. Eng. Geol..

[38] Piao C.D., Shi B., Wei G.Q., Yu L., Chen E.Y. (2015). BOTDA distributed measurement and analysis of mining overburden separation. J. Min. Saf. Eng..

[39] Sun B.Y., Zhang P.S., Fu M.R. (2022). Comparative study on the “optic-electric” monitoring method for the deformation and failure of surrounding rock in stopes. Nat. Hazards.

[40] Liu Y., Li W.P., He J.H., Liu S.W., Cai L.Y., Cheng G. (2018). Application of Brillouin optical time domain reflectometry to dynamic monitoring of overburden deformation and failure caused by underground mining. Int. J. Rock Mech. Min. Sci..

[41] Meng F.F., Piao C.D., Shi B., Sasaoka T., Shimada H. (2021). Calculation model of overburden subsidence in mined-out area based on Brillouin optical time-domain reflectometer technology. Int. J. Rock Mech. Min. Sci..

[42] Karhmis M., Triplett T., Haycocks C., Goodman G. (1983). Mining subsidence and its prediction in appalachian coalfield. Rock Mechanics: Theory, Experiment, Practice, Proceedings of the 24th US Symposium Rock Mechanics, College Station, TX, USA, 20–23 June 1983.

[43] Palchik V. (2002). Influence of physical characteristics of weal rock mass on height of caved zone over abandoned subsurface coal mines. Environ. Geol..

[44] State Administration of Safety Supervision, State Administration of Coal Mine Safety Supervision, State Energy Administration, State Railway Administration (2017). Specification for Coal Pillar Setting and Mining of Buildings, Water Bodies, Railways and Main Roadways.

[45] Li Q.S., Li X.B., Xu J.L., Xu Z.H., Zhang C. (2022). Research advances in mining fractures evolution law of rock strata and ecological treatment technology. Coal Sci. Technol..

[46] Chai J., Qiu B., Li Y., Zhu L. (2012). Simulation experiment of embedded fiber Bragg grating monitoring in rock deformation through borehole. J. Min. Saf. Eng..

[47] Wang H.Q., Zhu J.J., Fu H.Q., Feng G.C., Wang C.C. (2019). Modeling and robust estimation for the residual motion error in airborne sar interferometry. IEEE Geosci. Remote Sens. Lett..

[48] Xie H.P., Zhou H.W., Wang J.A., Li L.Z., Kwasniewski M.A. (1999). Application of FLAC to predict ground surface displacement due to coal extraction and its comparative analysis. Chin. J. Rock Mech. Eng..

[49] Xu Y.C., Zhang Y.Z. (2002). Deformation characteristics of the thick unconsolidated layers due to mining by UDEC. J. China Coal Soc..

[50] Zhang P.S., Liu S.D., Shu Y.F. (2011). Analysis on dynamic testing results of distortion and collapsing of the top rock by geophysical method during mining of coal seam. J. China Coal Soc..

[51] Ou Y.C., Zhang P.S., Wang W. (2019). Study on the evolution rule of land damage based on electrical resistivity imaging technology in mining face. Geotech. Geol. Eng..

[52] Cheng G., Wang Z.X., Shi B., Zhu H.H., Li G.Q., Tian L.Q. (2023). Research on multi-field fiber optic neural sensing and safety guarantee system constructing for mining overburden deformation. Coal Sci. Technol..

